# An Adaptable Quality Control Framework for Platelet-Rich Plasma as a Blood-Derived Product: A Multicenter Real-World Study

**DOI:** 10.3390/ph19101609

**Published:** 2026-10-10

**Authors:** Pedro Martí Picó, Diego Delgado, María Cayuela, Mikel Sánchez, Amrei Ewers, María Sanchez

**Affiliations:** 1 BioSmartData S.L., Carrer Xesc Forteza-Actor, 5, 07141 Marratxí, Spain; mcayuela@biosmartdata.eu (M.C.); aewers@biosmartdata.eu (A.E.); msanchez@biosmartdata.eu (M.S.); 2 Advanced Biological Therapy Unit, MiKS Hospital, C/Duque de Wellington, 33, 01010 Vitoria-Gasteiz, Spain; diego.delgado@hospitalmiks.com (D.D.); mikel.sanchez@hospitalmiks.com (M.S.)

**Keywords:** platelet-rich plasma, autologous platelet concentrate, quality control, critical quality attributes, quality by design, product-profile alignment, process variability, KOOS, real-world data

## Abstract

**Background and Objectives:** Platelet-rich plasma (PRP) composition and activity vary with patient-related factors, medication exposure, and preparation procedures. We developed an adaptable manufacturing quality control score (QC-Score) framework and retained clinical outcomes as a secondary exploratory layer. **Methods:** This retrospective multicenter study included 13,283 hematological analyses associated with administered infiltrations. Its product-profile alignment index (PPAI; 0–100) summarizes the alignment of platelet enrichment and residual white blood cell (WBC) and red blood cell (RBC) ratios with a study-defined compositional profile. The criteria are research proxies and not manufacturer or regulatory release limits. **Results:** The full PPAI was reportable in 8937 analyses and a platelet-only partial index in 404 analyses. The median full PPAI values for Systems A, B, and D were 74.6, 90.6, and 48.8, and complete-case conformance to the frozen study-defined benchmark was 9.8%, 15.8%, and 10.2%, respectively. System C was evaluated on platelet enrichment only. Modeling the recorded final-product WBC and RBC zeros as residual concentrations of 0.01 changed nine individual PPAI values but no system median or conformance count. Center–system patterns were heterogeneous. Secondary exploratory Knee Injury and Osteoarthritis Outcome Score (KOOS) analyses were sensitive to incomplete questionnaire handling and showed no consistent association with the PPAI. **Conclusions:** The QC-Score framework and PPAI support product characterization and targeted process investigation. Their operational proxies are neither manufacturer release limits nor regulatory specifications, and the PPAI was not validated as a clinical predictor.

## 1. Introduction

Platelet-rich plasma (PRP) is an autologous blood-derived preparation produced close to the point of care. Its composition and biological activity depend on the patient’s source blood and clinical context, as well as collection, separation technology, protocol selection, and local execution. Patient-related variability and medication exposure must therefore be considered when interpreting a preparation. Comparative studies have demonstrated substantial differences in platelet, leukocyte, and erythrocyte content across preparation methods and commercial systems, with additional donor and operator variability [1,2,3,4,5,6].

Cell counts also provide an incomplete account of platelet function. In a controlled laboratory study of healthy men, low-dose aspirin altered activation-dependent growth factor release without changing the platelet or leukocyte counts [7]. This finding illustrates a biological dimension that a cell count-based index cannot measure; it does not establish a clinical effect in the present cohort.

Existing characterization frameworks—including Platelets, Activation, and White Blood Cells (PAW); Platelets, Leukocytes, Red Blood Cells, and Activation (PLRA); Dose, Efficiency, Purity, and Activation (DEPA); and Minimum Information for Studies Evaluating Biologics in Orthopaedics (MIBO)—make PRP heterogeneity visible and improve compositional reporting [8,9,10,11,12]. They primarily classify or describe a preparation, however, and do not by themselves provide a routine process-monitoring method for asking whether observed measurements align with a study-defined product benchmark or which critical quality attribute (CQA) is responsible for a deviation. Incomplete preparation reporting remains common [13,14].

Quality by design (QbD) concepts provide an appropriate vocabulary for this problem: an intended product profile, measurable CQAs, risk-proportionate controls, and lifecycle learning [15,16,17]. These concepts were used solely as an analytical organizing framework; their application here does not imply good manufacturing practice manufacture, validated batch release, regulatory qualification, or a formal pharmaceutical quality system. An adaptable framework is required because a leukocyte-poor product with moderate platelet enrichment should not be judged against the same profile as a deliberately leukocyte-rich or highly concentrated preparation.

In this article, the QC-Score names the overall quality control framework; it reports what was measured, whether a sample can be assessed, how its composition aligns with the chosen profile, and how results vary across centers. The product-profile alignment index (PPAI) is its continuous 0–100 summary of platelet enrichment and residual white blood cell (WBC) and red blood cell (RBC) ratios. Higher values indicate closer alignment with the profile chosen for this study. They do not indicate the probability of clinical benefit, the percentage of pure platelets, or suitability for clinical use. Box 1 illustrates how to read the PPAI for an individual sample.

Box 1 **Reading PPAI for one sample.**

Consider three hypothetical samples with WBC and RBC ratios of 0.02 and 0.005, respectively, both within the study proxies. With all other inputs fixed, platelet enrichments of approximately 1.593-, 1.317-, and 1.117-fold produce full PPAI values of approximately 90, 70, and 50 under the formula in Methods, respectively. The decreasing score reflects increasing departure from the study’s 2–3-fold platelet target. Even the sample scoring 90 fails that platelet criterion. These are illustrative calculations and not observed patients or validated high, medium, or low categories. Different combinations of attributes can produce the same score; the components must always be read alongside it. System C’s platelet-only partial index has a different scope and should be read separately.

Manufacturing alignment and clinical response are distinct questions. A technically aligned product does not guarantee a benefit, and a technically non-aligned product does not establish treatment failure. The Knee Injury and Osteoarthritis Outcome Score (KOOS) was therefore designated as a secondary, exploratory clinical layer and was not used to define or tune the PPAI.

Using a governed real-world data infrastructure, this study transformed more than 13,000 routine hematological analyses into an auditable framework for detecting product variability, characterization limitations, and potential process deviations.

The primary objective was to develop and apply an adaptable QC-Score framework for PRP manufacturing in a large retrospective multicenter cohort. The secondary objective was to explore whether visit-specific cumulative product-profile alignment was associated with longitudinal KOOS outcomes in a linked knee-osteoarthritis subcohort.

## 2. Results

### 2.1. Manufacturing Cohort and Evaluability

The study included 13,283 hematological analyses associated with administered infiltrations from 5339 patients, 6275 prescriptions, and 35 pseudonymized private clinical centers in Spain. The analytical unit was one hematological analysis associated with one administered infiltration and not a verified regulatory batch. The age at the first dated analysis in the fixed cohort was calculable for 2212/5339 patients (41.4%); the median age in that available case subset was 56.1 years (interquartile range [IQR] = 44.4–67.3). The recorded gender was male for 2796 patients (52.4%), female for 2542 (47.6%), and “both” for 1 (<0.1%). Age was unavailable or invalid for 3127/5339 patients (58.6%); the available case age summary does not describe all participants.

Systems A, B, C, and D contributed 289, 10,350, 753, and 117 analyses, respectively. System B accounted for 77.9% of the full cohort and 89.9% of the mapped analyses. The corresponding numbers of contributing centers were 7, 13, 14, and 4, respectively; a center could contribute more than one system. Two centers contributed 9034/10,350 System B analyses (87.3%; Supplementary Table S3). This documents the concentration of recorded activity in a few centers, rather than balanced enrollment across systems. Longer data recording histories at some clinics using System B also contributed to its larger accumulated sample. The separate contributions of the recording duration and activity volume were not quantified. A further 1774 analyses were classified as UNMAPPED, meaning that the recorded product information was absent, unrecognized, or insufficient for a unique assignment. The reconciliation is explicit: 289+10,350+753+117+1774=13,283.

The PPAI was fully evaluable for 8937 analyses and partially evaluable for 404. The remaining 3942 were not scored: 1774 unmapped analyses, 1528 with another documented non-evaluable reason, 638 with a physically inconsistent volume, and 2 with an invalid denominator; 1774+1528+638+2=3942. The 1528 “other” records comprised 1357 nonpositive enrichment records, 138 retained deleted-source entities, and 33 duplicate active sample types after hierarchical assignment. Raw data quality flags can overlap, whereas this classification is mutually exclusive. No record was removed from the 13,283-analysis cohort flow because it showed low enrichment or failed a criterion. Score reportability and complete case conformance nevertheless required the fixed rules for whether a sample could be assessed; 1415 analyses had non-positive platelet enrichment values used for scoring outside the PPAI’s logarithmic domain. Consequently, the primary conformance rates were conditional on eligibility for score calculation. Table 1 and Figure 1 show the inclusive flow; Supplementary Figure S1 and Tables S1 and S2 provide the detailed evaluability and data quality audits.

### 2.2. QC-Score Framework, PPAI, and Study-Profile Conformance

The framework reports the PPAI together with its component attributes and the number of samples that can be assessed. The PPAI was reportable for 9341 analyses: 8937 full three-component indices and 404 platelet-only partial indices for System C.

For System A, the PPAI was evaluable in 235 analyses and had a median of 74.6 (interquartile range [IQR] = 6.1–95.8); 23/235 analyses met every active study criterion (9.8%; patient cluster bootstrap 95% confidence interval [CI] = 5.1–15.3%). System B had a median PPAI of 90.6 (IQR = 73.9–99.0), and 1364/8614 met all criteria (15.8%; 95% CI = 15.0–16.7%). System D had a median PPAI of 48.8 (IQR = 36.7–90.7), with 9/88 conforming (10.2%; 95% CI = 1.3–20.8%). System C was evaluated separately because the exact kit and preparation/output profile were unavailable; 404/753 analyses had a reportable platelet-only partial index, whereas 232/753 met the platelet criterion (30.8%; 95% CI = 27.3–34.5%). The criterion denominator includes all observed enrichment values; the partial index denominator additionally applied the fixed rules for whether a score could be calculated. No complete conformance rate was calculated for System C.

Table 2 and Figure 2 separate the full continuous index from each binary CQA. A high continuous score and binary non-conformance are not contradictory. For example, an analysis can remain close to the benchmark and retain a high PPAI while a white blood cell (WBC) ratio of 0.051 narrowly exceeds the frozen 0.05 proxy.

The low complete conformance proportions primarily reflect the conjunction of strict study-defined proxies and must not be interpreted as evidence of poor product quality, regulatory non-compliance, or device failure. System-level summaries are descriptive; no between-system hypothesis test or superiority comparison was performed because the sample sizes, center distributions, and evaluability differed markedly.

### 2.3. Center–System Variability

Center–system combinations showed heterogeneous PPAI and CQA profiles. The display rule in Figure 3 required at least 30 analyses and 20 patients. Within System B, complete conformance ranged from 8.8% in C01 to 35.6% in C17 among the displayed groups. The dominant deviation differed by center, supporting attribute-specific process investigation rather than a center or device ranking. Supplementary Table S3 provides the complete aggregate center–system summary.

The earlier QC-Score included a center-level consistency component inside each individual score. In the revised framework, consistency and process variability are analyzed separately, because a patient-level product measurement should not change merely because it was produced at a center with more or less historical dispersion.

### 2.4. Sensitivity Analyses and Measurement Limitations

System summaries were broadly stable after excluding revised records and under alternative component weights. Complete conformance depended more strongly on the chosen residual cell proxies; relaxing the WBC and RBC ratios from 0.05 and 0.01 to 0.10 and 0.02 changed rates from 9.8%, 15.8%, and 10.2% to 21.7%, 24.2%, and 19.3% for Systems A, B, and D, respectively. These scenarios illustrate dependence on the study profile and were not selected to obtain favorable rates.

The recorded final-product WBC or RBC zeros occurred in 3913/13,283 analyses (29.5%). Source checks supported retaining these numeric results but did not establish biological absence or PRP-specific analytical sensitivity. Replacing each recorded zero concentration with the minimum observed positive value, that being 0.01 in the corresponding analyte unit, changed nine individual PPAI values, no system median, and no complete conformance count. Removing all zero-bearing analyses materially changed the selected sample and was retained only as an extreme selection sensitivity.

Supplementary Figures S2–S4 and Tables S4–S8 and S13–S17 retain the detailed sensitivity and analyzer context evidence, including alternative denominators, the stored enrichment fallback, and hypothetical residual cell concentrations. The exact analyzer model and PRP-specific measurement performance could not be established for individual records.

### 2.5. Secondary Exploratory KOOS Results

Of 5269 KOOS visits from 1284 knee osteoarthritis episodes, 403 selected visits from 151 patients met the frozen timing and completion rules. In the fully evaluable single-system cohort, the paired pain changes at M3, M6, and M12 were 27.2, 30.6, and 35.9 points (*n* = 46, 55, and 27, respectively, all with p<0.001), and the KOOS4 changes were 26.2, 29.1, and 33.4 points (*n* = 43, 52, and 26, respectively, all with p<0.001). These estimates were materially attenuated or reversed when recorded-zero visits were retained. The continuous pain and KOOS4 models showed no consistent PPAI association, and the leave-one-center-out estimates changed signs. The clinical analysis was exploratory and did not validate the PPAI as a predictor. Supplementary Figures S5–S8 and Tables S9–S12, S18 and S19 provide the clinical flow, longitudinal outcomes, sensitivity analyses, models, and diagnostics.

## 3. Discussion

This study converts routine paired hematological measurements into a QC-Score framework that reports a continuous product-profile index, each component CQA, evaluability, and complete study-profile conformance. The principal contribution is a traceable process-monitoring view and not a device ranking or a clinical decision rule.

Three findings are most relevant to practice: compositional assessment was incomplete for a substantial part of the routine dataset; a high continuous alignment score could coexist with failure of a strict individual criterion; and the attributes responsible for deviation differed across the center–system groups. The practical next step is to examine the contributing measurements and local workflow. The score alone cannot identify the cause of a deviation or determine treatment suitability.

Continuous alignment and binary conformance answer different questions. The PPAI describes the distance from the common study benchmark; complete conformance requires every active proxy to be met. A result immediately above one proxy can therefore have a high PPAI but fail the binary conjunction. The reported primary rates are more precisely complete-case conformance because they are conditional on PPAI reportability; the broader all-observable-CQA sensitivity gave similar aggregate rates but a lower value for System D. Neither estimate is evidence that a nonconforming administered product was unusable or that a device failed.

The PPAI does not replace component-level CQA reporting. It provides a single continuous measure for monitoring distributions, screening center–system patterns, and prioritizing process investigations, whereas the individual platelet, WBC, and RBC components remain necessary to identify the attribute driving a deviation.

Primary documents supported the recorded product families but did not establish identical numerical WBC or RBC limits for every system [18,19,20,21,22]. The 0.05 and 0.01 thresholds therefore remain common study-defined proxies rather than manufacturer or regulatory specifications. System C could be identified only as the Fidia Hy-Tissue family; neither an exact kit nor a leukocyte profile was imputed.

The inclusive cohort is a methodological strength. It was compared with a historical internal analytical dataset that had excluded enrichment values below one. This unpublished dataset was used only to quantify the effect of the earlier selection rule and was not treated as an independent study. Here, low-alignment, incomplete, and non-evaluable observations were retained in the cohort flow, although non-positive enrichment remained outside the frozen logarithmic PPAI domain. Reporting the all-observable-CQA sensitivity makes this distinction explicit. Relaxing the WBC and RBC proxies increased complete conformance but did not eliminate the main finding of heterogeneous, attribute-specific deviations.

Center–system heterogeneity supports local review of training, protocol execution, sampling, and measurement workflows. It cannot be attributed solely to the device architecture because systems were unevenly distributed, several center–system cells were sparse, and process-parameter completeness was limited. The variability layer should therefore be used as an investigation signal rather than a league table.

Analyzer evidence remains an important limitation. The Mindray BC-20s and Dymind DH36 manuals describe impedance measurements for WBCs, RBCs, and platelets but do not establish PRP-specific analytical validation [23,24]. The analyzer model, operating mode, reportable ranges, flags, and PRP-specific validation were unavailable. Published blood component studies likewise show that low residual cell enumeration requires matrix- and mode-specific validation and that routine analyzers may be unsuitable for residual WBC measurement [25,26]. Variation in stored sender metadata and recorded-zero patterns may reflect technical or workflow factors, but the available evidence cannot identify the analyzer responsible for sender-missing messages or support device comparisons. The recorded zeros were therefore retained as observed and interpreted as compatible with unquantified low residual concentrations. The 0.01 sensitivity preserved every system median and conformance count; complete record exclusion was retained only as an extreme, non-random selection scenario.

The clinical layer was secondary and exploratory. Observed KOOS improvement cannot be attributed to PRP or to a PPAI value because there was no comparator, and the follow-up was select and incomplete. Sensitivity to questionnaire handling and unstable center sensitivity further limit interpretation. The PPAI is not a validated clinical predictor and should not guide individual treatment decisions.

Patient-related variation is a competing explanation for compositional differences. Dividing product cell counts by the patient’s baseline counts accounts for those measured starting concentrations; it does not adjust for comorbidities, inflammatory state, medication exposure, platelet function, or differences in treatment indication. Treatment indications and PRP preparation were recorded in routine care, but concomitant medication and comorbidities were not recorded systematically. These exposures could not be characterized reliably from the available records and were not included in the reported analyses. Diagnostic and preparation fields do not provide adjustment for these unmeasured factors. The aspirin laboratory findings illustrate how function can change while counts remain similar [7]; they do not justify attributing observed center differences to medication or changing a patient’s treatment. Because System B supplied most observations, and two centers supplied most of its records, the pooled estimates mainly reflect unequal recording durations and concentrated activity and should not be treated as population-wide or system-comparative estimates.

Future versions could add the final volume and total platelet dose (product platelet concentration multiplied by administered product volume, with explicit unit conversion), activation method and timing, select growth factor or platelet function measurements, and elapsed collection-to-preparation and preparation-to-administration times. The dose and volume would be reported first alongside the component measures; activation is a categorical process descriptor, and the preparation time is a potential process parameter and not automatically a scored CQA. Any additional desirability function, weight, or limit would require a stated biological rationale, suitable measurement validation, and prospective evaluation. Patient diagnoses, comorbidities, and time-linked medication exposure should accompany this characterization as contextual covariates. The current three-component PPAI is not a comprehensive biological potency or safety assessment.

Several limitations remain. This was a retrospective routine-practice study; the analytical unit was an analysis associated with an infiltration rather than a verified physical batch. Product identity was incomplete for 1774 analyses, the exact System C kit and preparation/output profile were unavailable, and several previous internal assumptions were not supported quantitatively by the primary documents reviewed. The stored platelet enrichment factor is a potentially derived fallback rather than an independently validated measurement, although every reportable PPAI in this freeze used the directly computed ratio. The analyzer model, mode, flags, and PRP-specific analytical performance were not stored. The unavailable analyzer assignment and System C variant and profile were treated as irreducible evidence limitations, and no record-level identity was imputed. The common benchmark, desirability functions, σ, weights, and operational proxies were study-defined choices fixed for this analytical freeze; they were not prospectively registered, externally validated, or established as system-specific specifications. Participant ages and recorded genders were added through a dated linkage to current clinical attributes, rather than being part of the original fixed analytical dataset. Age was calculable for only 41.4% of the patients, and thus its available case summary may not represent the full cohort; the recorded gender was reported using the controlled source categories and was not reinterpreted as biological sex. The clinical follow-up was select and incomplete, as the 1-month window was not estimable after visit adjudication, binary event cells were sparse, and leave-one-center-out analyses showed coefficient sensitivity. These constraints define the framework as an auditable research tool rather than a release specification.

## 4. Materials and Methods

### 4.1. Study Design and Data Sources

This retrospective multicenter observational study used pseudonymized laboratory, treatment, and patient-reported outcome records collected during routine clinical practice in private clinical centers in Spain. The manufacturing projection covered 13,283 analyses, of which 11,528 had recorded analysis dates from 21 October 2021 through the frozen cutoff of 6 April 2026; 1755 analysis dates were missing. It combined fixed extracts of routine laboratory and clinical records, with a separately linked longitudinal outcome dataset. The country and private clinic setting were confirmed by the responsible data controller. Participant ages and recorded genders were added on 31 July 2026 by linking the fixed patient cohort to current clinical attributes without changing the source records. Each patient contributed once to these summaries. The age at the first dated frozen analysis was retained only when it was between 0 and 120 years. The controlled recorded gender categories were male, female, and both; the male code was also the source default. The results therefore describe the recorded categories without assuming biological sex or independently verified capture. Treatment indications and PRP preparation were recorded in routine care. Concomitant medication and comorbidities were not recorded systematically by the participating clinics and could not be characterized reliably from the available records; their effects were not evaluated in the reported analyses. A separate historical export availability check is described in the Supplementary Materials. Patient-level data remained restricted, and only aggregate findings were used in this manuscript. Reporting was informed by the Strengthening the Reporting of Observational Studies in Epidemiology (STROBE) statement [27].

This study was conducted within BioSmartData S.L., which maintains the governed dataset, data infrastructure, and analytical environment used for this work. Routine-practice data were generated at the participating clinical centers. BioSmartData-affiliated authors participated in the study conception, data curation, analysis, interpretation, and manuscript preparation; individual contributions are reported in the author contributions statement.

Data checks used read-only access and did not alter any clinical records. Technical source linkage and measurement details are retained in the Supplementary Materials. The cohort reflects observed routine-practice records rather than balanced allocation to systems; no study quota was imposed to equalize the system counts. Some clinics using System B had been recording routine data for longer, contributing to its larger accumulated sample.

Generative artificial intelligence tools (OpenAI ChatGPT 5.6, accessed July–August 2026) were used only to support searches for the relevant literature and the drafting and language editing of the manuscript. Candidate sources identified with this assistance were checked by the authors against the underlying publications and were not treated as evidence without author verification.

### 4.2. Manufacturing Inclusion, Linkage, and Evaluability

An analysis entered the inclusive cohort when a documented infiltration could be linked to its treatment and prescription. Cohort inclusion was not conditional on enrichment, the PPAI, WBCs, RBCs, or conformance. Each retained record was then assigned exactly one evaluability category in this priority order: unmapped product; duplicate active sample type, retained deleted-source entity, or non-positive platelet enrichment value used for scoring grouped as another documented reason; invalid denominator; physically inconsistent volume; missing active CQA; partial evaluation; or full evaluation. This priority explains why overlapping raw flags do not sum to the mutually exclusive flow. UNMAPPED denotes absent, unrecognized, or insufficient product information for a unique A–D assignment.

Linked product and blood analysis context was used where available. Comparing records with richer linked information against laboratory records alone was a descriptive sensitivity and did not replace the inclusive cohort.

### 4.3. Product Mapping and Primary Evidence Audit

The recorded product identifiers mapped Arthrex autologous conditioned plasma (ACP), BTI Endoret plasma rich in growth factors (PRGF), the Fidia Hy-Tissue PRP family, and OLIN-1 to Systems A–D, respectively. These letters are neutral analytical labels and not an attempt to conceal the product families or imply blinded comparisons: the correspondence is explicitly disclosed in Table 3. Product names document provenance, while consistent letter labels keep the presentation focused on the analytical profiles. The systems were not randomly allocated, equally represented, or balanced for patients and centers; the results are descriptive and do not support a commercial ranking or superiority inference.

Four evidence layers were separated: a manufacturer- or team-described expected profile; a study-defined numerical proxy; an observed hematological value or product-to-baseline ratio; and the resulting operational conformance indicator. Primary manufacturer documents were reviewed for this revision [18,19,20,21]. Claims not located in those sources were downgraded to study-defined assumptions rather than presented as specifications.

### 4.4. Hematological Measurements and Analyzer Context

Platelet, WBC, and RBC measurements were obtained from paired baseline blood and final product samples. The product and baseline concentrations used the same documented unit within each analyte pair, making the ratios dimensionless. A value recorded as zero was retained as a measurement record and was not interpreted as proven cellular absence.

The supplied Mindray BC-20s and Dymind DH36 operator manuals describe impedance counting but do not establish PRP-specific analytical validation [23,24]. The exact analyzer assignment, operating mode, analytical flags, and PRP-specific performance were unavailable for individual records. No analyzer-specific correction or censoring rule was applied. Supplementary Table S16 describes the available evidence and its limits.

### 4.5. Critical Quality Attributes and Operational Profile

Within the QbD vocabulary, a CQA is a measurable product property relevant to the intended quality profile [15]. Here, three cellular attributes were selected as candidate CQAs for describing a moderate-enrichment, low residual cell profile; enrichment quantifies the concentration relative to source blood, while residual WBC and RBC ratios describe the cellular composition after separation. Their selection does not demonstrate clinical criticality or establish a complete pharmaceutical control strategy. This study did not assess every potentially relevant property, such as potency, activation, sterility, or stability.

Table 4 summarizes the study-selected compositional profile. Designating the platelet concentration as PLT, the scored attributes were platelet enrichment

$$E={\mathrm{PLT}}_{product}/{\mathrm{PLT}}_{baseline},$$

the residual leukocyte ratio

$$W={\mathrm{WBC}}_{product}/{\mathrm{WBC}}_{baseline},$$

and the residual erythrocyte ratio

$$R={\mathrm{RBC}}_{product}/{\mathrm{RBC}}_{baseline}.$$

When the product and positive-baseline platelet concentrations were both available, *E* was calculated directly. Otherwise, the stored dimensionless platelet enrichment factor was used as a fallback. This field may itself be derived, and it was not treated as an independent measurement. Direct calculation therefore had precedence, and a computation-only sensitivity was reported. The final product volume and platelet dose were descriptive and did not contribute to the PPAI.

The WBC and RBC thresholds are study-defined operational proxies and not manufacturer-validated release limits or regulatory release specifications. The 2–3-fold plateau represents the moderate platelet enrichment profile selected for this analysis. The 0.50, 0.30, and 0.20 weights encode the investigators’ analytical priority ordering—platelet enrichment first, followed by WBC and RBC reduction—rather than estimated effect sizes. The value σ=0.35 controls how rapidly platelet desirability decays on the log scale outside the target interval and is a tuning constant and not an estimated variance. The WBC and RBC ratios of 0.05 and 0.01 encode strict operational reductions of 95% and 99% relative to the baseline, respectively. These choices were fixed in the author-approved analytical design dated 20 July 2026, before the present device source audit and manuscript revision but after the retrospective data existed. They were not prospectively registered, optimized against conformance or KOOS, or externally validated. A common benchmark was applied to A, B, and D for comparability; it does not assert each manufacturer’s intended profile. The weight and threshold sensitivities are reported in Supplementary Figures S3 and S4, Tables S5 and S6.

### 4.6. Calculation and Interpretation of PPAI

The PPAI assigns each attribute a desirability between zero and one and combines the active components with a weighted geometric mean. This reduces compensation for one poorly aligned attribute by another well-aligned attribute. The construction follows the multi-response desirability approach of Derringer and Suich; that precedent does not validate the selected PRP targets or weights [28]. Center-level variability is reported separately. The earlier internal QC-Score formulation and the reason for this separation are documented in the Supplementary Materials.

Platelet enrichment desirability equals one within the 2.0–3.0 range and is penalized outside the nearest boundary using σ=0.35:

(1) $$d_{E}\left(E\right)=\left\{\begin{array}{cc} 1, & 2.0\leq E\leq 3.0,\\ \exp\;\left[-\frac{1}{2}{\left(\frac{\ln(E/2.0)}{0.35}\right)}^{2}\right],\; & E<2.0,\\ \exp\;\left[-\frac{1}{2}{\left(\frac{\ln(E/3.0)}{0.35}\right)}^{2}\right],\; & E>3.0. \end{array}\right.$$

For the upper-limit CQAs, we have

(2) $$d_{W}\left(W\right)=\left\{\begin{array}{cc} 1, & W\leq 0.05,\\ 0.05/W, & W>0.05, \end{array}\right.\;d_{R}\left(R\right)=\left\{\begin{array}{cc} 1, & R\leq 0.01,\\ 0.01/R, & R>0.01. \end{array}\right.$$

Under these frozen functions, a recorded W=0 or R=0 mechanically receives a desirability of one and meets its upper-limit proxy. This is an operational treatment of the recorded numeric value and not a statement of biological absence. For fully evaluable systems, we have

(3) $$\mathrm{PPAI}=100\exp\;\left[0.50\ln\left(d_{E}\right)+0.30\ln\left(d_{W}\right)+0.20\ln\left(d_{R}\right)\right].$$

For platelet-only System C, we have

(4) $${\mathrm{PPAI}}_{partial}=100d_{E}.$$

The partial index was not treated as dimensionally equivalent to the full three-component index.

### 4.7. Statistical Analysis of Manufacturing Measurements

The unit of analysis was a hematological analysis associated with an administered infiltration; patients could contribute repeated analyses. Continuous variables were summarized with medians and interquartile ranges, and categorical variables were summarized as counts with explicit denominators and percentages. The participant characteristics were summarized once per patient. No demographic imputation was performed.

Each component criterion used its own observable denominator. Complete conformance required every active criterion and was calculated among fully evaluable, PPAI-reportable analyses. System C contributed only platelet conformance and a separately labeled platelet-only partial index. For the primary conformance proportions, the 95% confidence intervals were the 2.5th and 97.5th percentiles of 5000 patient-cluster bootstrap replicates; all analyses from a sampled patient were retained together. This accounted for repeated analyses within a patient but did not remove between-center confounding. System comparisons were descriptive, with no between-system hypothesis test or superiority analysis.

Sensitivity analyses examined revised records, weights, residual-cell proxies, source completeness, the historical inclusion rule, direct versus stored enrichment, broader observable attribute denominators, and recorded-zero handling. Source data and the main analysis rules were retained. Detailed definitions and the software environments are provided in the Supplementary Materials; no scenario was selected on the basis of favorable conformance or a clinical association.

### 4.8. Secondary Exploratory Clinical Analysis

The clinical layer was restricted to knee osteoarthritis episodes assessed with KOOS [29]. A recorded pretreatment baseline was required. The fixed follow-up windows were 14–60, 61–135, 136–274, and 275–548 days for months 1, 3, 6, and 12, respectively. When more than one visit fell within a window, the visit closest to the nominal time was selected without inspecting its score. Under the investigator-adjudicated whole-visit rule, a visit in which every recorded native KOOS subscale was zero was excluded as a non-completed questionnaire; isolated or mixed zeros were retained. Missing follow-ups were not imputed, and alternative zero policies remained as sensitivity analyses.

Pain was the primary endpoint within this secondary exploratory layer; KOOS4 was a secondary, non-validated summary for this population. Paired baseline–follow-up changes were assessed by two-sided paired *t*-tests reported with their denominators and 95% confidence intervals. These change *p* values were exploratory and unadjusted for multiplicity; they did not test whether the PPAI predicted improvement.

At each follow-up, the cumulative PPAI was the arithmetic mean of evaluable infiltrations administered strictly before the assessment, excluding same-day and future infiltrations. The full-index cohort required a consistent A, B, or D system, known treatment dates, and full PPAI coverage for prior infiltrations. Longitudinal mixed-effects models included the baseline score, follow-up window, cumulative PPAI per 10 points, PPAI-by-window interaction, and system, with a patient random intercept. Patient-clustered binomial generalized estimating equations (GEEs) were used for operational minimal clinically important difference (MCID) and patient acceptable symptom state (PASS) endpoints when estimable. Benjamini–Hochberg correction was applied to PPAI hypotheses for the other native subscales, as detailed in the Supplementary Materials. Medication and comorbidity effects were not included in these models. Diagnostic checks, sparse event cells, missing follow-ups, and the leave-one-center-out sensitivity are reported in Tables S11–S12 and S19. The PPAI targets, weights, and limits were not fitted to KOOS outcomes.

## 5. Conclusions

The QC-Score framework organizes routine PRP measurements into a continuous alignment index, its component attributes, assessment coverage, and center–system variability. Its application to 13,283 analyses showed heterogeneous compositional profiles and highlighted the importance of reporting both the score and the individual criteria. The PPAI describes alignment with a study-defined profile; it is a research and process-monitoring tool and not a validated clinical predictor or a regulatory release test. The secondary KOOS findings do not support causal efficacy claims or individual treatment decisions.

Future work should prospectively standardize PRP sampling and analyzer validation, document patient conditions and medication exposure, and evaluate the dose, final volume, activation, growth factor or platelet function measures, and preparation timing. Any expanded index should use justified, prospectively specified criteria and undergo external evaluation while preserving separate component reporting.

## Figures and Tables

**Figure 1:**
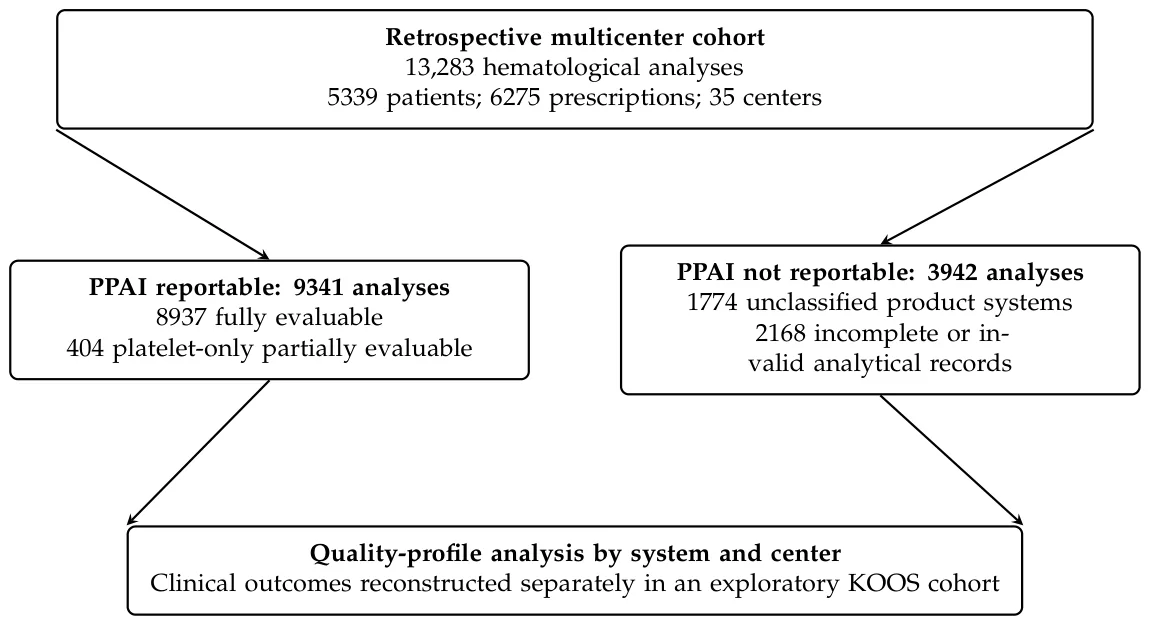
Manufacturing cohort and mutually exclusive evaluability classification. Clinical outcomes were reconstructed separately.

**Figure 2:**
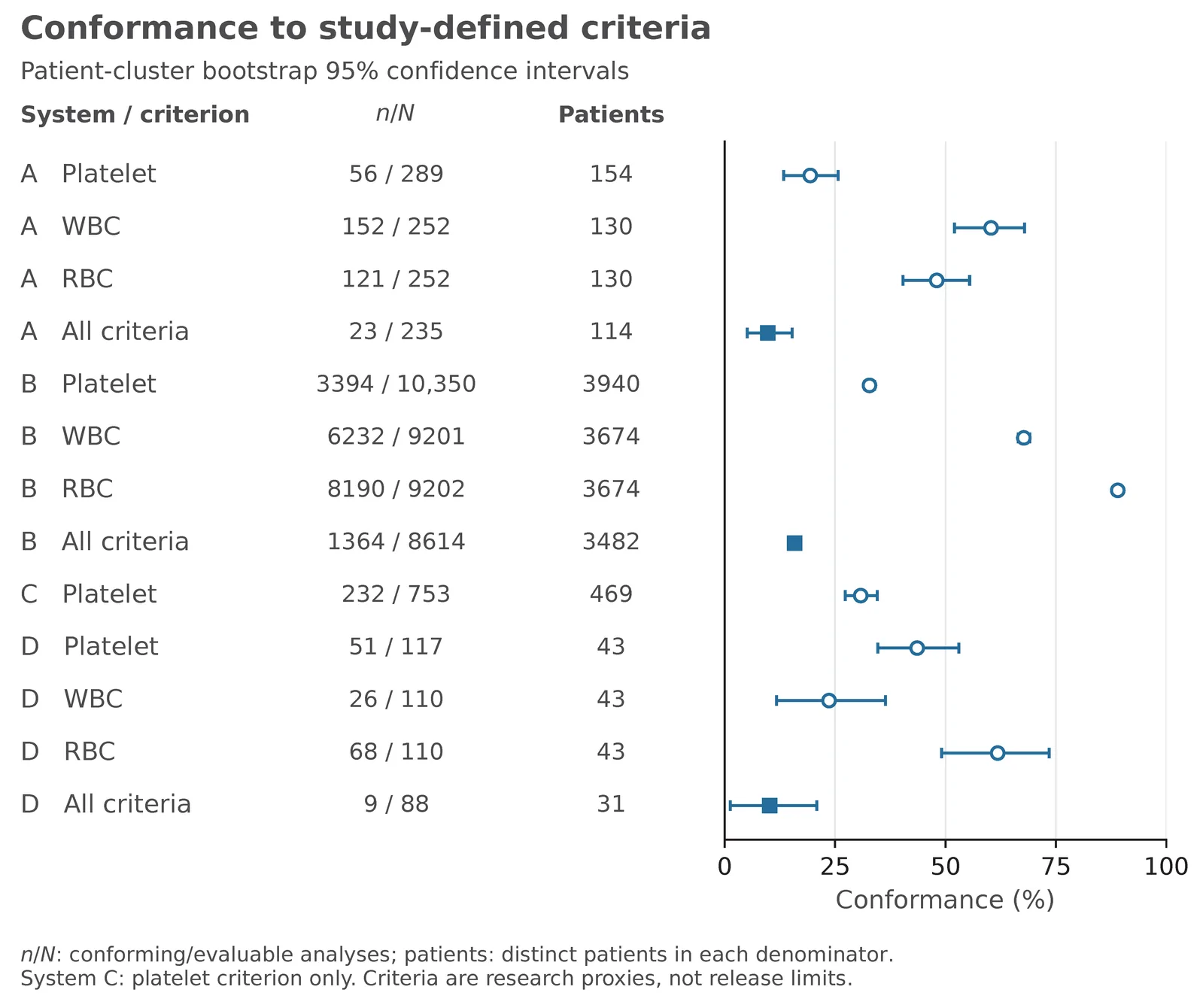
System-level CQA and complete study-profile conformance. Open circles indicate individual CQA criteria; filled squares indicate all active criteria jointly. Confidence intervals resampled patients while retaining all analyses contributed by each sampled patient. System C is shown only for platelet conformance, and its partial index is not pooled with the full PPAI.

**Figure 3:**
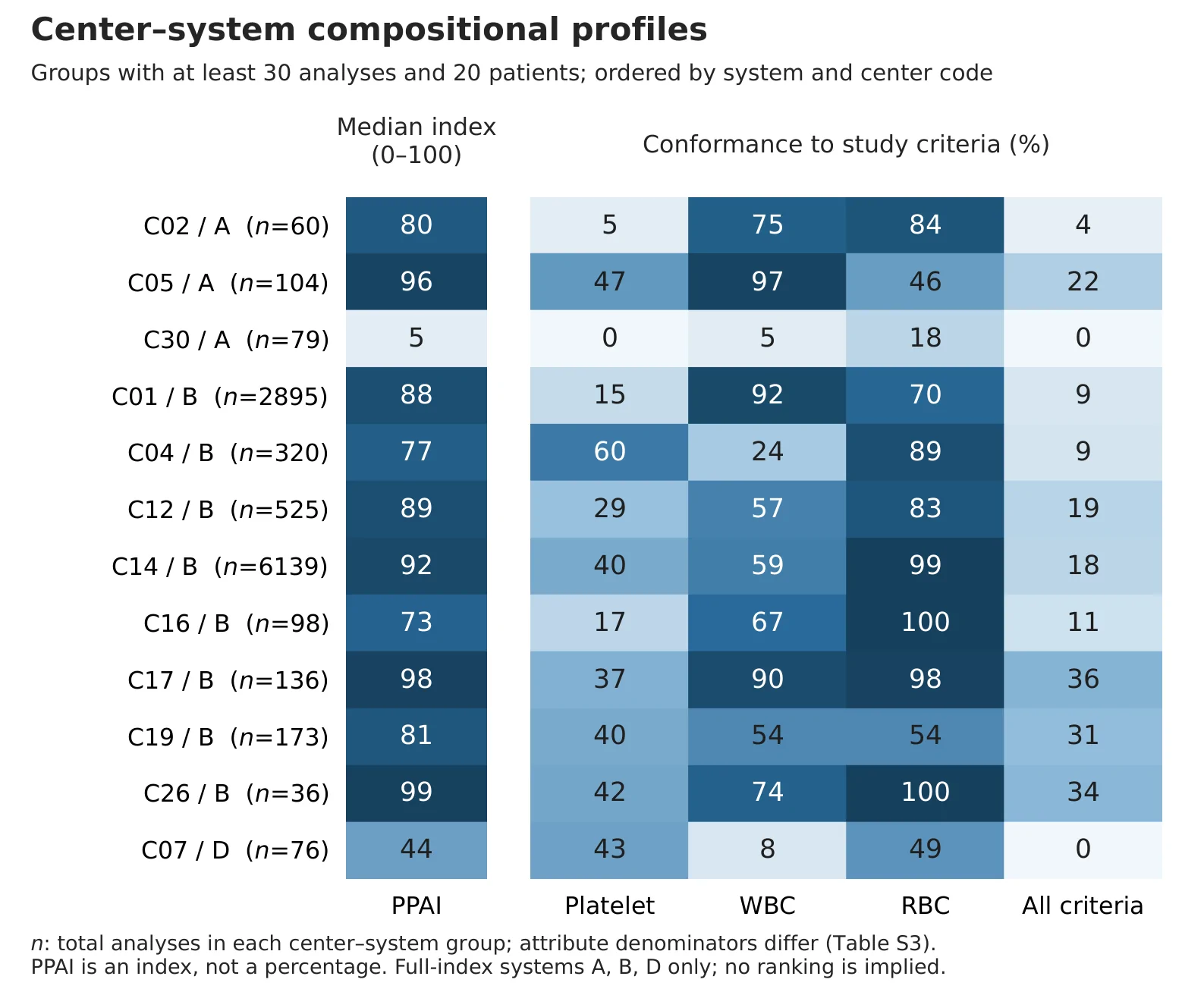
Product-profile patterns for center–system groups with at least 30 analyses and 20 patients. Values are PPAI medians or CQA or complete conformance percentages. Darker blue indicates higher values on a common 0–100 color scale; PPAI is an index, whereas conformance is a percentage.

**Table 1:** Manufacturing cohort and mutually exclusive evaluability classification.

Component	N	Interpretation
Hematological analyses	13,283	One analysis associated with one administered infiltration
Patients	5339	Unique patients in the manufacturing cohort
Prescriptions	6275	One or more administered infiltrations
Pseudonymized centers	35	Private clinical centers in Spain
Valid age at first dated analysis	2212	41.4% of patients; median 56.1 years [IQR = 44.4–67.3]
Unavailable or invalid age	3127	58.6% of the 5339 patients
Recorded gender	5339	Male 2796; female 2542; both 1
Fully evaluable	8937	Platelet, WBC, and RBC components available
Partially evaluable	404	Platelet component only
UNMAPPED	1774	Product information insufficient for a unique system assignment
Other documented non-evaluable reason	1528	Retained in the cohort flow but not scored
Physically inconsistent volume	638	Retained and flagged as non-evaluable
Invalid denominator	2	Product-to-baseline ratio not mathematically evaluable

Age and recorded gender were linked on 31 July 2026 to the fixed cohort and summarized once per patient. Gender describes the source field; the male category also served as a source default, and thus a populated field does not prove explicit ascertainment. IQR = interquartile range; WBC = white blood cell; RBC = red blood cell.

**Table 2:** PPAI and study-defined operational conformance by system.

Metric	System A	System B	System D
Analyses	289	10,350	117
Full PPAI, *n*	235	8614	88
PPAI median [IQR]	74.6 [6.1–95.8]	90.6 [73.9–99.0]	48.8 [36.7–90.7]
Platelet criterion, n/N (%)	56/289 (19.4)	3394/10,350 (32.8)	51/117 (43.6)
WBC criterion, n/N (%)	152/252 (60.3)	6232/9201 (67.7)	26/110 (23.6)
RBC criterion, n/N (%)	121/252 (48.0)	8190/9202 (89.0)	68/110 (61.8)
All active criteria, n/N (%) [95% CI]	23/235 (9.8) [5.1–15.3]	1364/8614 (15.8) [15.0–16.7]	9/88 (10.2) [1.3–20.8]

**Interpretation:** All criteria are study-defined research proxies. They are neither manufacturer release specifications nor regulatory limits. Failure to meet this combined study profile does not establish device failure or clinical unsuitability. System C is assessed for platelets only and is not included in the full PPAI or complete conformance.

**Table 3:** Product evidence and implications for the coded analytical profiles.

System	Recorded Product Family	Primary Evidence Located	Analytical Treatment
A	Arthrex ACP	2–3-fold platelet profile; technical data report reduced WBC and RBC counts [18]	Full PPAI; common 0.05 and 0.01 proxies remain study-defined and are not identical to the cited reductions
B	BTI Endoret/PRGF	Optimal platelet concentration; insignificant or no leukocytes; no erythrocytes [19]	Full PPAI; numeric common proxies remain study-defined
C	Fidia Hy-Tissue PRP family; exact kit and preparation profile unavailable	Manufacturer describes both extremely pure and leukocyte-rich outputs [20]	Platelet-only partial PPAI; no WBC or RBC evaluation
D	OLIN-1	Closed system and PRP above baseline; no quantitative WBC or RBC reduction located [21]	Full PPAI under study-defined common proxies; no manufacturer specification claim

**Table 4:** Study-selected candidate critical quality attributes and operational profile.

Attribute	Variable	Study Profile or Role	Interpretation
Platelet enrichment	*E*	2.0–3.0; range desirability; weight: 0.50	Concentration relative to source blood
Residual leukocytes	*W*	≤0.05; upper-limit desirability; weight: 0.30	Study-defined residual WBC profile
Residual erythrocytes	*R*	≤0.01; upper-limit desirability; weight: 0.20	Study-defined residual RBC profile
Final product volume	mL	Descriptive; not scored	Characterization and consistency check
Platelet dose	Total platelets	Descriptive; not scored	Product concentration multiplied by volume, with unit conversion

## Data Availability

The dataset presented in this article is not readily available because it contains internal BioSmartData information subject to confidentiality restrictions. The underlying dataset cannot be shared. Requests for aggregated study metrics may be directed to the corresponding author and will be considered in accordance with BioSmartData’s governance procedures.

## Supplementary Materials

The following supporting information can be downloaded at https://www.mdpi.com/article/10.3390/ph19101609/s1. Figure S1: Evaluability by system. Figure S2: Revised-record sensitivity. Figure S3: Weight sensitivity. Figure S4: Threshold sensitivity. Figure S5: KOOS zero-policy sensitivity. Figure S6: PPAI clinical associations. Figure S7: Clinical episode eligibility. Figure S8: KOOS longitudinal change. Table S1: Data quality audit. Table S2: Evaluability breakdown. Table S3: Center–system summary. Table S4: Update reason sensitivity. Table S5: Weight sensitivity. Table S6: Threshold sensitivity. Table S7: Historical selection impact. Table S8: Clustered intervals. Table S9a–d: Clinical eligibility, zero-pattern, zero-scenario, and baseline availability audits. Table S10: Longitudinal outcomes. Table S11a–g: Exploratory clinical models and diagnostics. Table S12a,b: Missing follow-up and threshold reference audits. Table S13: Measurement contract sensitivities. Table S14: Product cell zero sensitivities. Table S15: Graded product cell zero sensitivity. Table S16: Analyzer-context evidence. Table S17: Combined graded product cell zero sensitivity (summary of Table S15). Table S18: Paired longitudinal outcomes (summary of Table S10). Table S19: Aggregate leave-one-center-out sensitivity (summary of Table S11g). Tables S16–S19 are embedded in the supplementary PDF; Tables S1–S15, including lettered sub-tables, are supplied as CSV files. The PDF also contains a glossary and expanded technical methods. The sources cited in the supplementary methods are also included in the reference list here [23,24,28,29,30,31,32].

## Abbreviations

The following abbreviations are used in this manuscript:

ACP	Autologous conditioned plasma
CI	Confidence interval
CQA	Critical quality attribute
GEE	Generalized estimating equation
DEPA	Dose, Efficiency, Purity, and Activation
GDPR	General Data Protection Regulation
HL7	Health Level Seven
IQR	Interquartile range
KOOS	Knee Injury and Osteoarthritis Outcome Score
MCID	Minimal clinically important difference
PASS	Patient acceptable symptom state
MIBO	Minimum Information for Studies Evaluating Biologics in Orthopaedics
PAW	Platelets, Activation, and White Blood Cells
PLRA	Platelets, Leukocytes, Red Blood Cells, and Activation
PLT	Platelet concentration
QbD	Quality by design
QC-Score	Quality Control Score
PPAI	Product profile alignment index
PRP	Platelet-rich plasma
PRGF	Plasma rich in growth factors
RBC	Red blood cell
SQL	Structured Query Language
STROBE	Strengthening the Reporting of Observational Studies in Epidemiology
WBC	White blood cell
