# Supplementary material for: An Adaptable Quality Control Framework for Platelet-Rich Plasma as a Blood-Derived Product: A Multicenter Real-World Study
Source: Pharmaceuticals (Basel). 2026 Oct 10;19(10):1609. doi: 10.3390/ph19101609 (PMC13708724; doi:10.3390/ph19101609)
Supplement: Supplementary file 1 [file pharmaceuticals-19-01609-s001.zip › Supplementary_Material.pdf]

Supplementary Materials: An Adaptable Quality Control Framework for Platelet-Rich Plasma as a Blood-Derived Product: A Multicenter Real-World Study

Pedro Martí Picó, Diego Delgado, María Cayuela, Mikel Sánchez, Amrei Ewers, and María Sanchez

1. Manufacturing Evaluability and Sensitivity

Figures S1–S4 show the complete manufacturing evaluability and sensitivity analyses. These analyses retain the frozen PPAI formula and do not select assumptions on the basis of a favorable conformance rate.

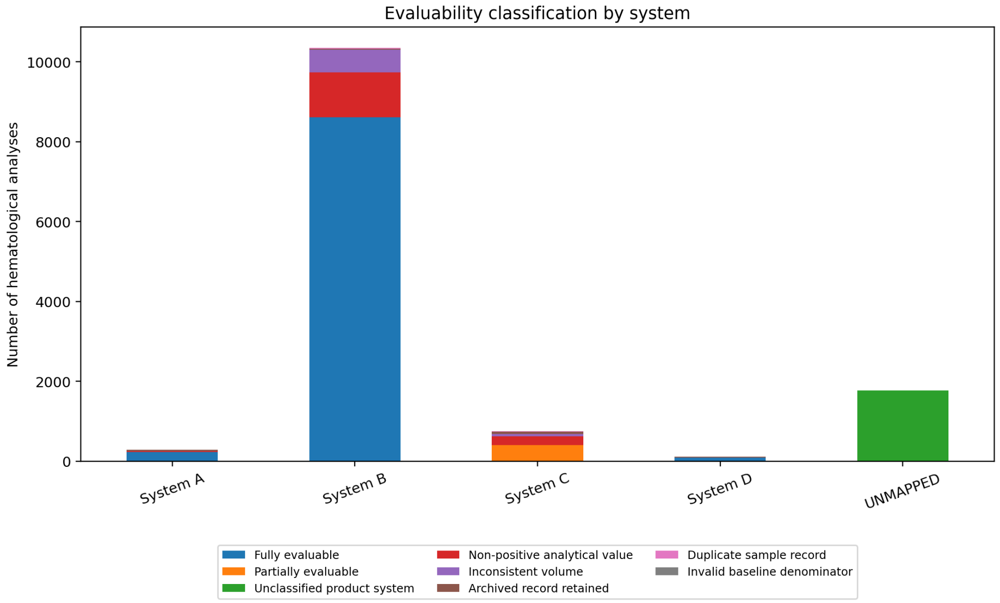

Figure S1. Mutually exclusive evaluability classification by system.

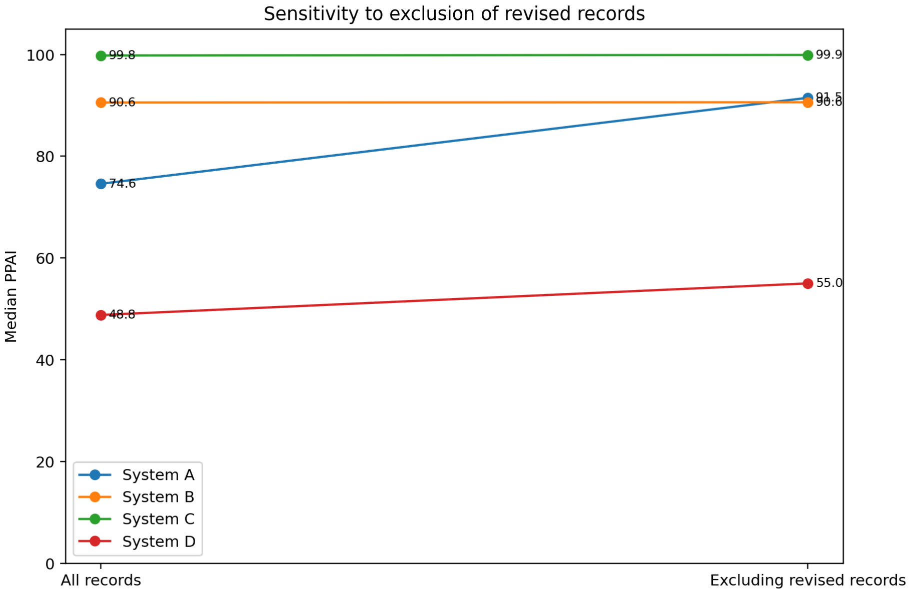

Figure S2. PPAI sensitivity after excluding records with documented revisions.

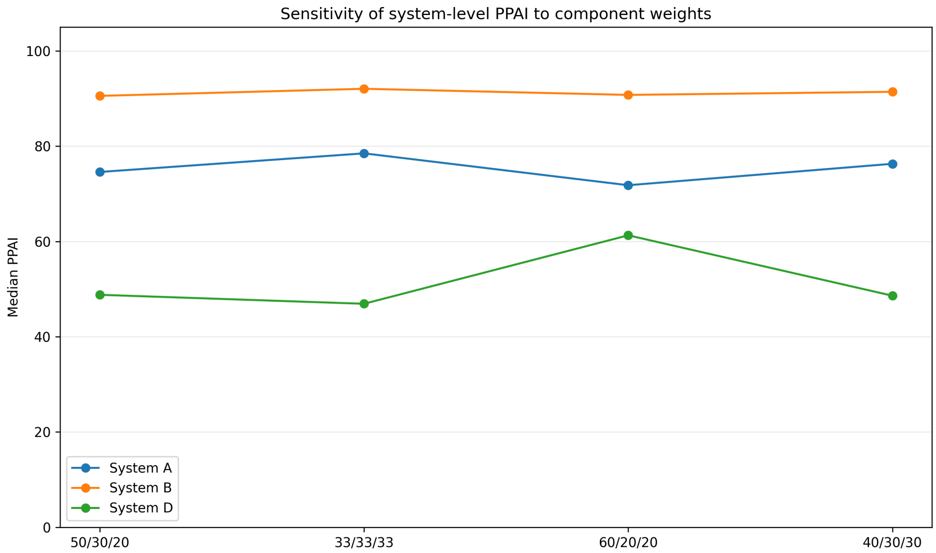

Figure S3. Sensitivity of system-level median PPAI to alternative component weights.

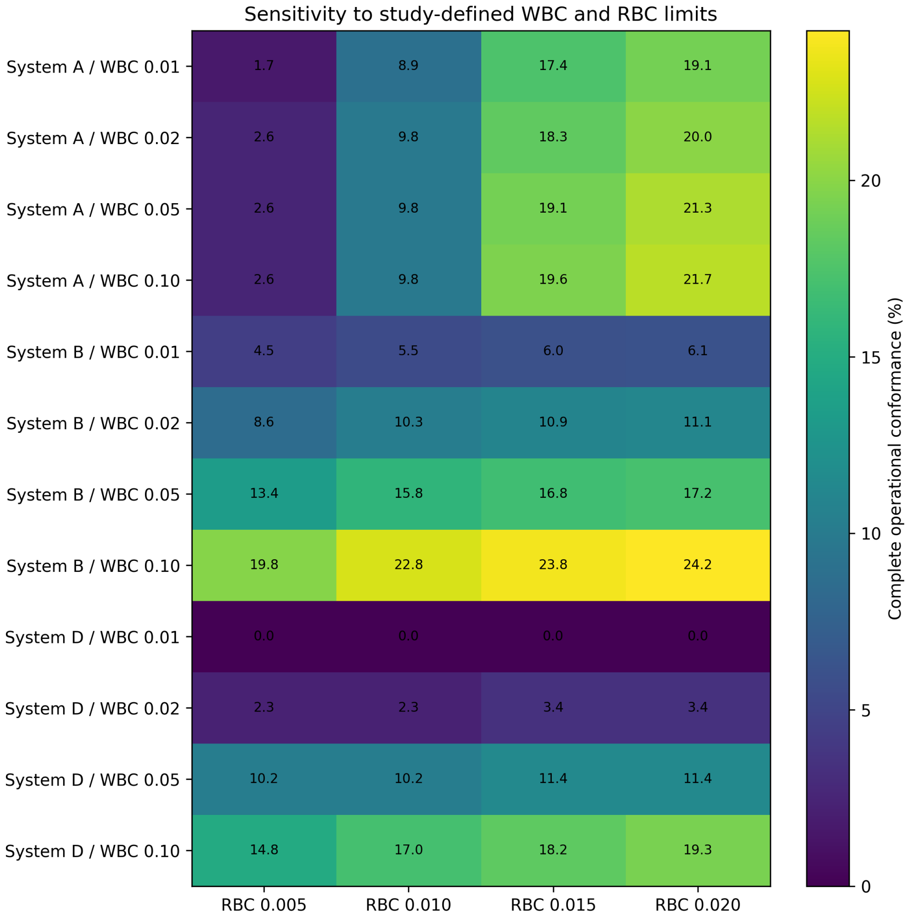

Figure S4. Complete operational conformance across the frozen WBC/RBC proxy grid. The scenarios are study-defined and are not manufacturer release limits.

2. Glossary and Reading Guide

QC-Score and PPAI.

QC-Score is the overall quality-control framework; the Product-Profile Alignment Index (PPAI) is its continuous 0–100 measure of alignment with the study profile.

Critical quality attribute (CQA).
A measurable product property selected for assessment against an intended profile. The three cellular attributes here are study-selected candidates, not validated clinical or regulatory release criteria.

Evaluability contract / frozen reportability.
Fixed rules specifying the identity, measurements, and data-quality checks required before a sample can receive a score. A sample that cannot be scored is still counted in the cohort flow.

Active enrichment.
The platelet product-to-baseline ratio used by the analysis, with a stored-factor fallback when direct calculation was unavailable. The stored factor is not an independent validation measurement.

Product-cell zeros.
Final-product WBC or RBC values recorded numerically as zero. They are not evidence of biological absence or a validated analytical detection limit.

Study-profile conformance.
All applicable binary study criteria are met. This is distinct from a continuous PPAI value and from clinical or regulatory acceptance.

HL7, SQL, and MongoDB.
Health Level Seven (HL7) refers to laboratory message exchange; Structured Query Language (SQL) is used to query relational records; MongoDB is the document database used for linked clinical context. These describe data provenance, not biological measurements or score components.

Bootstrap, CI, and IQR.
A bootstrap resamples observations to estimate uncertainty; here, patients were resampled with all their analyses. CI denotes confidence interval and IQR the middle 50% of observed values.

KOOS.
Knee injury and Osteoarthritis Outcome Score, used only in the secondary exploratory clinical layer. Months are labeled M1, M3, M6, and M12.

3. Detailed Manufacturing Sensitivity and Source Audit

Results were broadly stable when records with documented revisions were excluded and when alternative frozen component weights were applied. Complete conformance was more sensitive to the WBC and red blood cell (RBC) proxies: under the frozen 0.05/0.01 limits it was 9.8%, 15.8%, and 10.2% for Systems A, B, and D; under the less stringent 0.10/0.02 sensitivity it was 21.7%, 24.2%, and 19.3%. Rates therefore changed materially but remained low because complete conformance required the conjunction of platelet, WBC, and RBC criteria. These scenarios were not chosen to increase the rates. Supplementary Figures S2–S4 and Tables S4–S8 report the revision, weight, threshold, historical-selection, and clustered-interval sensitivities.

Primary complete-case conformance was also compared with an all-observable-CQA denominator that counted nonpositive enrichment as failure and required numerical platelet, WBC, and RBC ratios but did not apply the PPAI, traceability, or volume-consistency gates. Rates were 26/252 (10.3%), 1,424/9,201 (15.5%), and 9/110 (8.2%) for Systems A, B, and D, compared with 23/235 (9.8%), 1,364/8,614 (15.8%), and 9/88 (10.2%) in the primary reportable denominator. Because the broader denominator includes records excluded by other frozen quality flags, it is a sensitivity analysis rather than a replacement estimand (Supplementary Table S13).

The stored `PlateletFactor` supplied enrichment as a fallback when the direct platelet product-to-baseline ratio was unavailable in 1,446/11,509 mapped analyses (12.6%). Of these fallbacks, 1,413 were zero. A computed-only sensitivity reproduced all reportable PPAI medians and primary conformance rates exactly because every reportable PPAI already used a direct ratio. It did change platelet-only descriptive coverage: for System C, for example, conformance was 232/753 (30.8%) under the frozen hierarchy versus 229/498 (46.0%) among direct ratios only (Supplementary Table S13).

Final-product WBC and RBC were recorded as zero in 3,095/13,283 (23.3%) and 1,514/13,283 (11.4%) analyses, respectively. Either product-cell value was zero in 3,913/13,283 (29.5%), including 696 analyses with both values recorded as zero. Read-only tracing found exact agreement with current SQL for all 11,492 comparable product-cell values and with MongoDB for all 9,309 overlapping values. Among strictly linked final-product HL7 records, 2,947/2,973 stored WBC zeros (99.1%) and 1,365/1,394 stored RBC zeros (97.9%) had only numerical zero messages; the remaining cases had both zero and positive repeat messages, never positive messages alone. The minimum positive HL7 value was 0.01 for both analytes. These findings support retaining zero as the recorded result while treating it as compatible with unquantified low residual concentrations, not as proof of biological absence.

In the primary minimum-observed-positive-value sensitivity, each recorded zero concentration was replaced by 0.01 before recomputing its product-to-baseline ratio; source data, PPAI weights, and operational proxies were unchanged. Among 8,937 fully evaluable A/B/D analyses, 3,515 contained at least one product-cell zero. Only nine individual PPAI values changed, no system median changed, and complete conformance remained 1,396/8,937 (15.6%): 23/235 for A, 1,364/8,614 for B, and 9/88 for D. The extreme exclusion scenario retained 5,422 analyses and changed the System A median from 74.6 to 10.2 because it removed 95/235 observations; it is therefore interpreted as a selection sensitivity, not as evidence that residual concentrations invalidate the primary score (Supplementary Table S14).

In the graded hypothetical sensitivity, assigning WBC zeros values from 0.01 to 0.10 while holding RBC zeros at 0.01 left the combined PPAI median at 90.24 and conformance at 1,396/8,937 (15.6%). At WBC values of 0.20 and 0.40, the median was 90.17 and 86.63 and conformance was 1,360/8,937 (15.2%) and 1,055/8,937 (11.8%), respectively. Holding WBC zeros at 0.01, RBC values of 0.05 and 0.10 yielded medians of 89.77 and 85.52 and conformance of 1,232/8,937 (13.8%) and 1,181/8,937 (13.2%). These investigator-defined scenarios are neither inferred residual concentrations nor analytical or regulatory limits (Supplementary Table S15).

The read-only audit found all 13,283 frozen analysis identifiers in the current Structured Query Language (SQL) source. A unique barcode-to-Health Level Seven (HL7) message link was available for 11,679 analyses: 4,981 linked only to messages declaring `SENDER=Dymind`, 6,697 linked only to messages without a sender, and one linked to both categories; 1,604 analyses lacked a unique link. Neither SQL nor MongoDB stored the exact analyzer model. Exact analyzer assignment was unavailable, and no analyzer-specific interpretation was attempted. Accordingly, the database evidence does not prove that Dymind messages came specifically from a DH36 or that sender-missing messages came from a Mindray BC-20s.

Table S16. Read-only hematology-analyzer context and evidence boundary.

Evidence layer	N	Available information	Interpretation
Frozen SQL analysis identifiers	13,283	Analysis, sample, treatment, prescription, and center linkage	All frozen identifiers remained present
Uniquely linked analyses	11,679	4,981 Dymind-only; 6,697 sender-missing; 1 mixed	Manufacturer-string evidence for a subset, not a model assignment
Frozen MongoDB blood-analysis documents	9,332	Cellular measurements; no manufacturer, model, unit, mode, or flags	Cannot identify the analyzer
Supplied operator manuals	2	Mindray BC-20s and Dymind DH36 technical characteristics	Candidate context; no database-backed center–model mapping

4. Technical Data Provenance and Measurement Methods

The archived source-verification audits used read-only access in July–August 2026. SQL queries comprised one `SELECT` statement, optionally preceded by common table expressions; the connection set `ApplicationIntent=ReadOnly`, disabled autocommit, and rolled back on closure. MongoDB access was restricted to read operations without `$out`, `$merge`, or server-side code. No database value, schema, status, or record was modified. No additional source extraction was performed for the present editorial revision.

Patient-context field availability.

A read-only availability audit during revision inspected a separate historical export containing 2,937 analyses from 1,469 patients. All analysis identifiers and their patient, prescription, treatment, and center links matched the fixed manufacturing cohort. The export documented selection filters, including a platelet factor of at least one; its field completeness cannot be generalized to the full 13,283-analysis cohort. Treatment-indication codes and blood/PRP volumes were populated for all 2,937 records, and centrifugation speed and duration fields for 2,552 (86.9%). Populated fields were not independently validated clinical or process assessments. No dedicated concomitant-medication history or comorbidity fields were present. An infiltration-medicine field belonged to the infiltration record and did not establish a time-linked systemic-medication history. The contraindication field contained zero in every row; this cannot establish verified absence of contraindications or comorbidities. The export was used only to check field availability and did not replace or enrich the frozen analytical dataset. Source files were preserved unchanged, and no model was refitted.

Platelet, WBC, and RBC measurements came from paired baseline-blood and final-product SQL sample columns whose field contract encoded the same unit for both members of a pair. MongoDB values were used for source reconciliation but carried no independent unit metadata. The SQL HL7 source contained sender, date, sample barcode, WBC, RBC, platelet, mean platelet volume, and configured unit fields, but not analyzer model, firmware, whole-blood/predilution mode, hematology flags, or quality-control/calibration status. MongoDB blood-analysis documents contained no analyzer-identification fields.

The two operator manuals supplied by the study team were reviewed as candidate technical context. The Mindray BC-20s and Dymind DH36 use electrical impedance for WBC, RBC, and platelet counting and are intended for whole-blood, capillary, or prediluted blood matrices [1,2]. Their background specifications are not limits of detection, limits of quantification, biological absence thresholds, or PRP validation. The database sender string `Dymind` was not promoted to a DH36 model assignment, and missing sender was

not promoted to BC-20s. Exact center–model mapping was unavailable; therefore, no analyzer-specific interpretation, correction, or censoring rule was applied.

5. Historical Internal QC-Score and Revised PPAI

The earlier internal QC-Score combined enrichment, purity, and center-level consistency arithmetically:

$$\text{QC-Score}_{earlier} = 0.40\,\text{Score}_E + 0.40\,\text{Score}_P + 0.20\,\text{Score}_C. \qquad \text{(S1)}$$

Its enrichment component peaked at 2.5-fold, purity combined WBC and RBC, and consistency used the center-level platelet coefficient of variation. The present framework retains the recognizable QC-Score name but calls the revised continuous index PPAI because the mathematical construct changed. PPAI gives each CQA an explicit desirability, uses a 2–3-fold plateau rather than a single optimum, uses a geometric mean to limit compensation, and moves center consistency outside the individual score. The weighted geometric construction follows the general multi-response desirability approach of Derringer and Suich, but that precedent does not validate the exact PPAI functions or parameters [3].

6. Additional Statistical and Software Details

Each CQA and complete conformance were reported separately. Primary complete-case conformance required every active criterion and was calculated only for fully evaluable, PPAI-reportable analyses. The all-observable-CQA denominator sensitivity required numerical enrichment, WBC, and RBC ratios; nonpositive enrichment was an observed platelet failure, while missing ratios remained non-evaluable. Continuous variables were summarized with medians and interquartile ranges. Because patients could contribute multiple analyses, primary conformance 95% confidence intervals used a patient-cluster percentile bootstrap with 5,000 replicates. Frozen sensitivities excluded revised records, varied component weights and WBC/RBC proxies, compared source tiers, compared the inclusive cohort with a historical internal selection-rule dataset, restricted enrichment to directly computed ratios, and broadened binary conformance to all observable CQAs. For the product-cell zero minimum-observed-positive-value sensitivity, each recorded final-product WBC or RBC zero was replaced by a concentration of 0.01—the minimum positive value observed in strictly linked HL7 messages—before recomputing the corresponding ratio and PPAI under the unchanged frozen formula. This scenario was a hypothetical sensitivity value, not an imputation or an analyzer-specific limit of quantification. A second, extreme selection sensitivity removed every fully evaluable A/B/D analysis containing either recorded zero and re-summarized the retained records without reweighting. Source data and the primary estimand remained unchanged. No scenario was selected on the basis of a favorable rate or clinical association. System-level comparisons were descriptive; no between-system hypothesis test was performed.

The manufacturing freeze was executed in Python 3.12.3 using NumPy 2.4.4 and pandas 3.0.2. The frozen clinical analyses used Python 3.12.3 with NumPy 2.3.2, pandas 2.3.2, SciPy 1.16.2, statsmodels 0.14.5, and Patsy 1.0.1; the authorized diagnostic refits used Python 3.11.9 with the same library versions. Matplotlib 3.10.8 was used to render the retained analytical figures. scikit-learn 1.6.1 was installed in the project environment but was not used for any analysis reported in this study.

7. Supplementary Clinical Methods

The clinical analysis was restricted to knee-osteoarthritis episodes assessed with KOOS [4]. Baseline was the first pre-treatment KOOS assessment recorded as the initial evaluation.

Episodes without a baseline or with a baseline dated after the first treatment were excluded. Follow-up windows were 14–60 days (M1), 61–135 days (M3), 136–274 days (M6), and 275–548 days (M12); when several visits occurred in a window, the visit closest to the nominal time was selected without inspecting scores.

A selected visit in which every recorded native KOOS subscale was exactly zero was classified as a non-completed questionnaire according to investigator confirmation of the clinical workflow on 17 July 2026 and excluded in its entirety. An isolated zero or a mixed zero/non-zero visit was retained because zero is a valid KOOS extreme. Missing visits were not imputed. The all-recorded-zero-visits-retained and individual-zero-outcome-rows-excluded scenarios were kept as required sensitivities.

Pain was the exploratory primary clinical endpoint. KOOS4 was a secondary summary calculated as the equal-weight mean of Pain, Symptoms, Sport/Recreation, and Quality of Life when all four were available. This historical composite omits Activities of Daily Living (ADL), but was not treated as a validated total score for knee osteoarthritis [5,6]. All five native subscales, including ADL, were reported separately. Minimal clinically important difference (MCID) and patient acceptable symptom state (PASS) were operational exploratory endpoints under the frozen threshold contract [7].

Paired baseline–follow-up changes were tested against zero using two-sided paired t tests within each domain and follow-up window. These p-values were exploratory and were not adjusted for multiplicity; exact values are provided in Table S10.

For each selected follow-up, cumulative PPAI was the arithmetic mean of non-missing PPAI values from infiltrations administered strictly before the assessment; same-day and future infiltrations were excluded. The primary A/B/D cohort required one consistent system, known treatment dates, complete PPAI coverage, and full evaluability of all prior infiltrations. Model coefficients scaled this mean per 10 PPAI points. Longitudinal mixed-effects models used a patient random intercept:

$$\begin{aligned}\text{Score}_{it} = {} & \beta_0 + \beta_1 \text{Baseline}_i + \beta_2 \text{Window}_t + \beta_3 \text{PPAI}_{it} \\ & + \beta_4 (\text{PPAI}_{it} \times \text{Window}_t) + \beta_5 \text{System}_i + u_i + \epsilon_{it}.\end{aligned} \tag{S2}$$

Patient-clustered binomial generalized estimating equations were used for MCID and PASS when estimable. Benjamini–Hochberg correction was applied to PPAI hypotheses for the other native subscales. No cut point, PPAI tercile, time window, formula, weight, or product proxy was optimized against clinical results.

8. Secondary Exploratory Clinical Analyses

Figures S5–S8 show the zero-policy sensitivity, PPAI model estimates, episode eligibility, and paired longitudinal change. The clinical layer is secondary and exploratory.

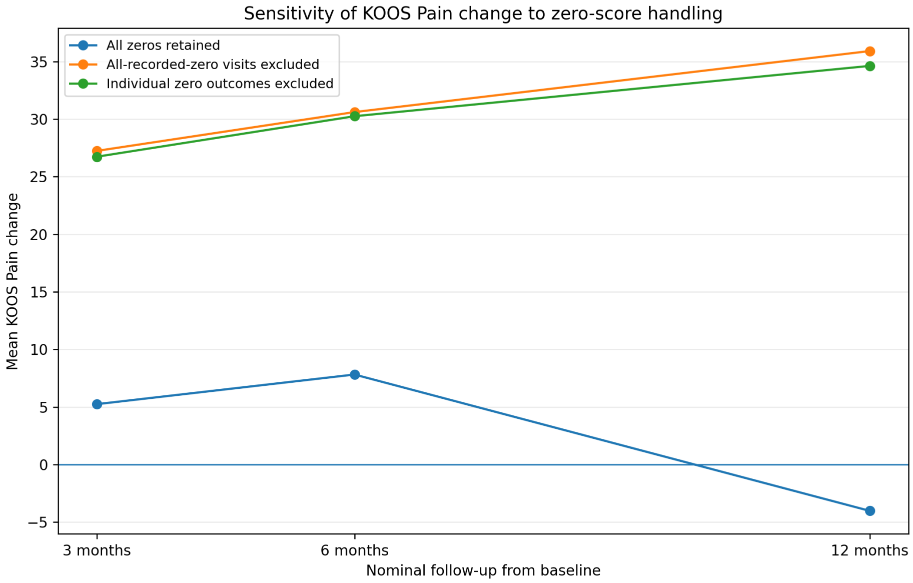

Figure S5. Sensitivity of mean KOOS Pain change to zero-score handling. Retaining all recorded-zero visits yielded changes of 5.2, 7.8, and −4.0 points at M3, M6, and M12; excluding whole all-recorded-zero visits yielded 27.2, 30.6, and 35.9; excluding individual zero outcome rows yielded 26.7, 30.2, and 34.6. The principal exploratory scenario uses the whole-visit rule.

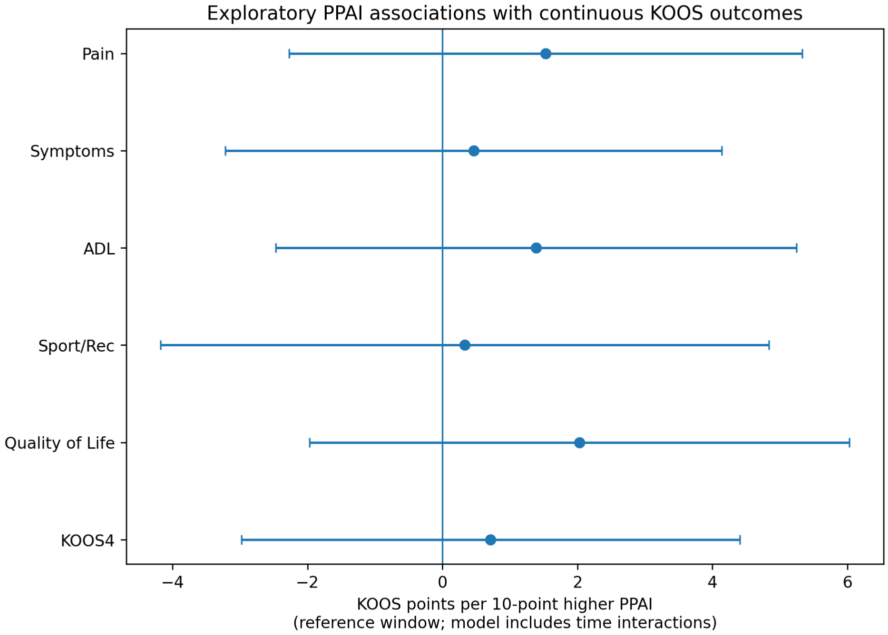

Figure S6. Exploratory reference-window PPAI coefficients for continuous KOOS outcomes. Models include PPAI-by-time interactions; the plotted term corresponds to the M12 reference window.

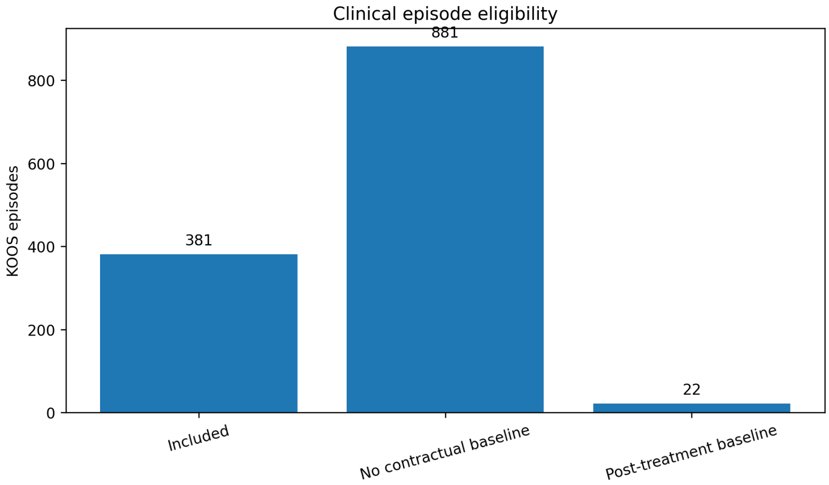

Figure S7. Clinical episode eligibility. Episodes without a recorded pre-treatment baseline or with a baseline dated after treatment were excluded explicitly.

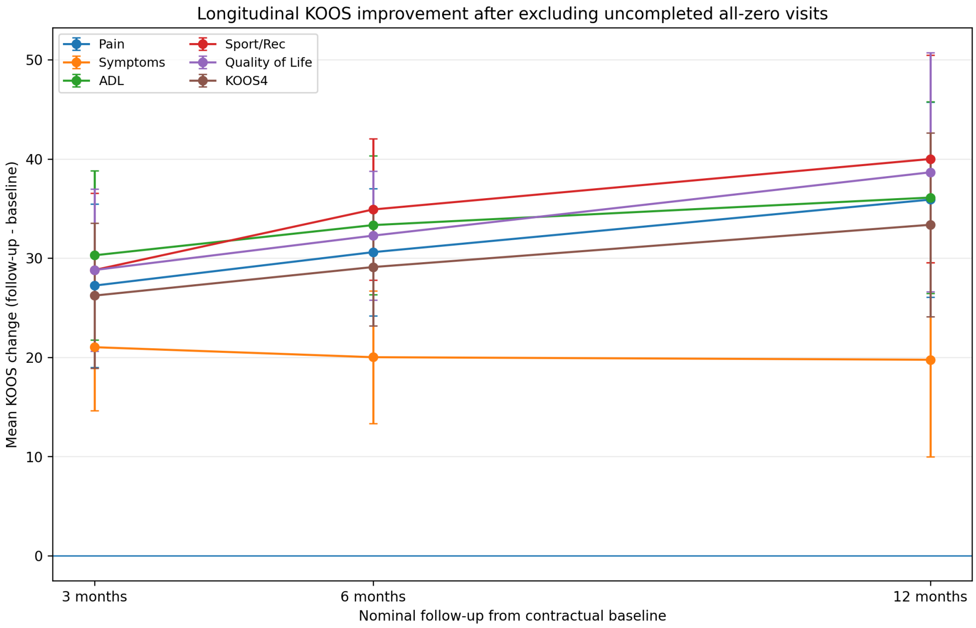

Figure S8. Mean paired change from baseline by KOOS domain and follow-up window. Error bars are 95% confidence intervals.

9. Accompanying Aggregate Tables

Tables S1–S15, including lettered sub-tables, are provided as accompanying CSV files, which can be opened in spreadsheet software. They contain no direct patient identifiers. This PDF contains Tables S16–S19. Table S16 describes analyzer-context evidence; Tables S17, S18, and S19 present summary views of CSV Tables S15, S10, and S11g, respectively. The distinct numbers identify these PDF summaries separately from the accompanying tabular files:

- Table S1: `Table_S1_Data_Quality_Audit.csv`;
- Table S2: `Table_S2_Evaluability_Breakdown.csv`;
- Table S3: `Table_S3_Centre_System_Summary.csv`;
- Table S4: `Table_S4_Update_Reason_Sensitivity.csv`;
- Table S5: `Table_S5_Weight_Sensitivity.csv`;
- Table S6: `Table_S6_Threshold_Sensitivity.csv`;

- Table S7: Table_S7_Historical_Selection_Impact.csv;
- Table S8: Table_S8_Clustered_Intervals.csv;
- Table S9a: Table_S9a_Episode_Eligibility.csv;
- Table S9b: Table_S9b_Zero_Patterns.csv;
- Table S9c: Table_S9c_Zero_Scenario_Flow.csv;
- Table S9d: Table_S9d_Baseline_Availability.csv;
- Table S10: Table_S10_Longitudinal_Outcomes.csv;
- Table S11a: Table_S11a_Pain_Model.csv;
- Table S11b: Table_S11b_KOOS4_Model.csv;
- Table S11c: Table_S11c_GEE_Models.csv;
- Table S11d: Table_S11d_Continuous_Diagnostics.csv;
- Table S11e: Table_S11e_Binary_Diagnostics.csv;
- Table S11f: Table_S11f_Binary_Event_Cells.csv;
- Table S11g: Table_S11g_Leave_One_Centre_Out.csv;
- Table S12a: Table_S12a_Missing_Followup.csv;
- Table S12b: Table_S12b_Threshold_Reference.csv;
- Table S13: Table_S13_Measurement_Contract_Sensitivities.csv;
- Table S14: Table_S14_Product_Cell_Zero_Sensitivities.csv;
- Table S15: Table_S15_Graded_Product_Cell_Zero_Sensitivity.csv.

Table S13 makes the conformance-denominator and enrichment-source measurement-contract sensitivities explicit. All-observable-CQA conformance counts nonpositive enrichment as failure and does not require reportable PPAI; rates are 10.3%, 15.5%, and 8.2% for A, B, and D. Although 12.6% of mapped enrichment values use the stored-factor fallback, all reportable PPAI values already use direct ratios, so computed-only PPAI results are unchanged.

Table S14 separates two questions. The minimum-observed-positive-value scenario replaces each recorded final-product WBC or RBC zero with a concentration of 0.01 before recomputing its product-to-baseline ratio under the unchanged PPAI formula. This changed only 9/8,937 individual PPAI values, no system median, and no conformance count: combined conformance remained 1,396/8,937 (15.6%). The value 0.01 was the minimum positive result observed in strictly linked HL7 messages and is used only as a hypothetical sensitivity value, not as an imputation or an analyzer-specific limit of quantification. The extreme exclusion scenario removes all 3,515 zero-bearing analyses, leaving 5,422 records; its large effect on the System A median reflects removal of 95/235 observations and is interpreted as non-random cohort selection rather than evidence that small residual concentrations invalidate the primary score.

10. Graded Product-Cell Zero Sensitivity

Table S15 reports an investigator-defined hypothetical concentration grid in which final-product WBC and RBC values recorded as zero were temporarily assigned positive concentrations before product-to-baseline ratios were recomputed under the unchanged PPAI formula. The WBC sweep assigned 0.01, 0.05, 0.10, 0.20, or 0.40 while holding RBC zeros at 0.01; the RBC sweep assigned 0.01, 0.02, 0.05, or 0.10 while holding WBC zeros at 0.01. The primary analysis continued to retain all zeros exactly as recorded.

At 0.01/0.01, the combined median was 90.24 and conformance remained 1,396/8,937 (15.62%). WBC assignments through 0.10 did not change either value; WBC assignments of 0.20 and 0.40 reduced conformance to 1,360/8,937 (15.22%) and 1,055/8,937 (11.80%). RBC assignments of 0.05 and 0.10 reduced conformance to 1,232/8,937 (13.79%) and 1,181/8,937 (13.21%). System-specific results and proxy-exceedance counts are retained in the accompanying CSV.

Every graded scenario is a hypothetical, investigator-defined analytical sensitivity. None is a primary imputation, an analyzer-specific or manufacturer-specified limit of quantification, a device specification, a validated release limit, or evidence that a particular residual cellular concentration was present.

Table S17 presents the combined A/B/D summary of the accompanying Table S15.

Table S17. Combined A/B/D graded sensitivity to hypothetical concentrations assigned only to final-product WBC or RBC values recorded as zero. All scenarios include 8,937 fully evaluable analyses; the primary source values remain unchanged.

Sweep	WBC zero	RBC zero	PPAI median	PPAI changed, n	Conformance, n (%)
WBC	0.01	0.01	90.24	9	1,396 (15.62)
WBC	0.05	0.01	90.24	13	1,396 (15.62)
WBC	0.10	0.01	90.24	22	1,396 (15.62)
WBC	0.20	0.01	90.17	184	1,360 (15.22)
WBC	0.40	0.01	86.63	2,210	1,055 (11.80)
RBC	0.01	0.01	90.24	9	1,396 (15.62)
RBC	0.01	0.02	90.24	10	1,395 (15.61)
RBC	0.01	0.05	89.77	1,171	1,232 (13.79)
RBC	0.01	0.10	85.52	1,399	1,181 (13.21)

11. Longitudinal KOOS Descriptives

Table S18 reports paired baseline and follow-up values for all native KOOS domains and KOOS4. Endpoint-specific denominators are preserved. Exact two-sided paired t-test p-values for change against zero are included in the accompanying `Table_S10_Longitudinal_Outcomes.csv` file.

Table S18. Paired longitudinal outcomes for the fully evaluable single-system A/B/D cohort after exclusion of investigator-adjudicated all-recorded-zero visits.

Domain	Window	N	Baseline	Follow-up	Change [95% CI]	MCID	PASS
Pain	M3	46	52.0	79.2	27.2 [19.0–35.5]	69.6%	71.7%
Pain	M6	55	50.3	80.9	30.6 [24.2–37.0]	90.9%	74.5%
Pain	M12	27	45.6	81.5	35.9 [26.1–45.7]	85.2%	77.8%
Symptoms	M3	44	54.0	75.0	21.0 [14.6–27.4]	68.2%	77.3%
Symptoms	M6	53	55.8	75.8	20.0 [13.3–26.7]	64.2%	75.5%
Symptoms	M12	26	49.4	69.1	19.8 [10.0–29.5]	57.7%	69.2%
Activities of Daily Living	M3	42	51.0	81.3	30.3 [21.8–38.8]	64.3%	69.0%
Activities of Daily Living	M6	51	49.9	83.2	33.3 [26.3–40.3]	86.3%	68.6%
Activities of Daily Living	M12	27	46.4	82.5	36.1 [26.4–45.8]	74.1%	70.4%
Sport/Recreation	M3	46	33.6	62.4	28.8 [21.0–36.6]	73.9%	54.3%
Sport/Recreation	M6	55	29.3	64.2	34.9 [27.8–42.0]	78.2%	65.5%
Sport/Recreation	M12	27	29.4	69.4	40.0 [29.5–50.5]	88.9%	66.7%
Quality of Life	M3	46	36.8	65.6	28.8 [20.6–37.0]	69.6%	52.2%
Quality of Life	M6	55	33.1	65.3	32.3 [25.8–38.8]	83.6%	50.9%
Quality of Life	M12	27	33.6	72.2	38.7 [26.6–50.7]	85.2%	66.7%
KOOS4	M3	43	43.8	70.0	26.2 [18.9–33.6]	–	–
KOOS4	M6	52	41.8	70.9	29.1 [23.2–35.0]	–	–
KOOS4	M12	26	39.0	72.3	33.4 [24.1–42.6]	–	–

12. Aggregate Leave-One-Center-Out Sensitivity

All six center omissions were estimable for Pain and KOOS4, but the PPAI estimate changed sign twice for each outcome. Omitted-center identities remain restricted. Table S19 presents the summary of the accompanying Table S11g.

Table S19. Aggregate leave-one-center-out sensitivity for continuous PPAI terms.

Outcome	Centers	Full estimate	Minimum	Maximum	Sign changes
Pain	6	1.531	-1.615	2.980	2
KOOS4	6	0.714	-3.031	3.030	2

13. Interpretation Contract

Manufacturer-described expected profiles, study-defined operational proxies, observed hematological results, and operational-conformance indicators are separate evidence layers. Recorded WBC/RBC zeros do not prove cellular absence even though the frozen numerical functions treat zero ratios as meeting upper-limit proxies. Primary complete-case conformance is conditional on PPAI reportability; the all-observable-CQA sensitivity uses a broader denominator. System C remains evaluable on platelet enrichment only and its partial index is not dimensionally comparable with full three-component PPAI. The secondary clinical analyses do not validate PPAI as a predictor or estimate causal treatment effectiveness.

References

1. Shenzhen Mindray Bio-Medical Electronics Co., Ltd.. *BC-20s Auto Hematology Analyzer Operator's Manual*. Mindray, Shenzhen, China, 2015. Issued February 2015; operator manual supplied to the study team.
2. Shenzhen Dymind Biotechnology Co., Ltd.. *DH36 Auto Hematology Analyzer Operator Manual*. Dymind, Shenzhen, China, 2016. Document S-65020020A[5.0]; operator manual supplied to the study team.
3. Derringer, G.; Suich, R. Simultaneous Optimization of Several Response Variables. *Journal of Quality Technology* **1980**, *12*, 214–219. https://doi.org/10.1080/00224065.1980.11980968.
4. Roos, E.M.; Lohmander, L.S. The Knee injury and Osteoarthritis Outcome Score (KOOS): From joint injury to osteoarthritis. *Health and Quality of Life Outcomes* **2003**, *1*, 64. https://doi.org/10.1186/1477-7525-1-64.
5. Frobell, R.B.; Roos, E.M.; Roos, H.P.; Ranstam, J.; Lohmander, L.S. A Randomized Trial of Treatment for Acute Anterior Cruciate Ligament Tears. *New England Journal of Medicine* **2010**, *363*, 331–342. https://doi.org/10.1056/NEJMoa0907797.
6. KOOS. Frequently Asked Questions: What Is KOOS4? https://www.koos.nu/faq.html, 2026. Official KOOS information resource; accessed 30 July 2026.
7. Boffa, A.; Andriolo, L.; Franceschini, M.; Di Martino, A.; Asunis, E.; Grassi, A.; Zaffagnini, S.; Filardo, G. Minimal Clinically Important Difference and Patient Acceptable Symptom State in Patients With Knee Osteoarthritis Treated With PRP Injection. *Orthopaedic Journal of Sports Medicine* **2021**, *9*, 23259671211026242. https://doi.org/10.1177/23259671211026242.
